# Influence of size and surface capping on photoluminescence and cytotoxicity of gold nanoparticles

**DOI:** 10.1007/s11051-018-4406-0

**Published:** 2018-11-15

**Authors:** Cecilia Fernández-Ponce, Juan P. Muñoz-Miranda, Desiré M. de los Santos, Enrique Aguado, Francisco García-Cozar, Rocío Litrán

**Affiliations:** 1 Department of Biomedicine, Biotechnology and Public Health, University of Cadiz, Cádiz, Spain; 2 Institute of Biomedical Research Cádiz (INIBICA), Cádiz, Spain; 3 Department of Physical Chemistry and Instituto de Microscopía Electronica y Materiales (IMEYMAT), University of Cádiz, Cádiz, Puerto Real Spain; 4 Department of Condensed Matter Physics and Instituto de Microscopía Electronica y Materiales (IMEYMAT), University of Cádiz, Cádiz, Puerto Real Spain

**Keywords:** Gold nanoparticles, Cytotoxicity, Cell viability, Proliferation, Photoluminescence, Capping, Agent, GSH, Semiconfocal microscopy

## Abstract

Hydrophilic and homogeneous sub-10 nm blue light-emitting gold nanoparticles (NPs) functionalized with different capping agents have been prepared by simple chemical routes. Structure, average, size, and surface characteristics of these NPs have been widely studied, and the stability of colloidal NP solutions at different pH values has been evaluated. Au NPs show blue PL emission, particularly in the GSH capped NPs, in which the thiol-metal core transference transitions considerably enhance the fluorescent emission. The influence of capping agent and NP size on cytotoxicity and on the fluorescent emission are analyzed and discussed in order to obtain Au NPs with suitable features for biomedical applications. Cytotoxicity of different types of gold NPs has been determined using NPs at high concentrations in both tumor cell lines and primary cells. All NPs used show high biocompatibility with low cytotoxicity even at high concentration, while Au-GSH NPs decrease viability and proliferation of both a tumor cell line and primary lymphocytes.

**Electronic supplementary material:**

The online version of this article (10.1007/s11051-018-4406-0) contains supplementary material, which is available to authorized users.

## Introduction

Gold nanoparticles (Au NPs) are interesting nanomaterials used in a wide variety of technologies, including catalysis (Peng et al. 2017; Wei et al. 2018; Zhu et al. 2010), nanoelectronics (Bafrani et al. 2018; Barman and Verma 2010; Dong et al. 2017; Huang et al. 2018; Jain et al. 2008; Kamyshny and Magdassi 2014), and biomedicine (Dreaden et al. 2012; Giljohann et al. 2010; Huang et al. 2009; Jain et al. 2008; Khan et al. 2018).

Over the last two decades, Au NPs have been mainly used as biosensors and as contrast agents in X-ray imaging and computed tomography (Kobayashi et al. 2016), being applied in biological and biomedical research, especially in cancer diagnosis (Chuang et al. 2018; Kim et al. 2018; Sugumaran et al. 2018) and therapy (Khandanlou et al. 2018; Zhu et al. 2018), biosensing (Baetsen-Young et al. 2018; Chen et al. 2009), virus detection, and regulation of cell function (Bodelon et al. 2017; Giljohann et al. 2010).

The biomedical interest of these NPs resides in their good biocompatibility, low inherent toxicity (Lewinski et al. 2008), facile synthesis (Jain et al. 2006), high biofunctionalization capability (Li et al. 2018), and especially, in their unique optical properties.

The so-called localized surface plasmon resonance (LSPR) (Hu et al. 2006) is a phenomenon that can be described as the collective coherent oscillation of conduction electrons with respect to a positive metallic lattice, occurring when small metallic NPs are stimulated by the electromagnetic field of incident light (Hutter and Fendler 2004). Consequently, these metallic NPs show a high absorption cross section in the visible-NIR range, making them appropriate agents for bioimaging and even for treatment by hyperthermia (Ghosh et al. 2008; Huang et al. 2009). In the case of gold, the LSPR band is located around 500–540 nm for NPs with sizes smaller than 30 nm. However, the LSPR band position is affected by size, shape, interparticle distance, and dielectric constant, as well as the refractive index of surrounding media, which can shift the LSPR, even to the NIR range (Haes et al. 2004; Jain et al. 2007; Sugumaran et al. 2018).

Absorption is not the only optical property that can make these NPs useful in bioimaging. Recently, photoluminescence (PL) of gold NPs has attracted considerable attention, due to their potential applications (Liu et al. 2014). Interband radiative recombination of electrons in noble metals was first reported by Mooradian in 1969 (Mooradian 1969), while Wilcoxon in 1998 observed a PL band around 440 nm in small gold clusters and proposed a mechanism for luminescence in Au nanoparticles (Wilcoxon et al. 1998). Afterwards, other groups have reported tunable emissions, from UV to NIR in gold NPs (Chen et al. 2009). The emission of these NPs is usually assigned to the interband transition Au 5d^10^ to 6sp and also to the capping ligand-metal charge transfer transition. Other authors have reported the influence of size and NP surface in the PL emission (Liu et al. 2014).

Functionalization of the NP surface with species containing bio-active terminal groups, such as amino or carboxylic groups, allows for a subsequent linking with relevant biomolecules (Albanese et al. 2012; Miao et al. 2018). NP functionalization can also contribute to avoiding aggregation and unspecific cellular uptake, minimizing accumulation in organs and/or phagocyte activation, thus maintaining a prolonged circulation time (Nel et al. 2009; Silvestri et al. 2017; Sperling and Parak 2010; Verma and Stellacci 2010). For biomedical applications, surface functionalization is a common strategy not only to subsequently promote cross-linking between NPs and specific biological species but also to minimize their cytotoxicity and unspecific binding.

Preparation methods that allow for a direct functionalization such as “Bottom-up” methods are the most common strategies for the preparation of monodispersed Au NPs (Huang et al. 2009). The first chemical synthesis route to prepare Au NPs was the Turkevich method (Turkevich et al. 1951), a reducing reaction that uses sodium citrate as reducing agent. This method has been modified for many different authors in order to increase the monodispersity of the resulting NPs (Piella et al. 2016; Zhao et al. 2012). Many other authors have reported on gold NPs prepared by reduction in the presence of a capping ligand that directly biofunctionalize the NP or facilitate its subsequent biofunctionalization (Fratoddi 2018), controlling the particle growth. This route also allows for the use of thiols as capping agents, due to the high affinity of this group for the gold surface (Brust et al. 1994; Dehn et al. 2018).

In this work, we have synthesized four different types of Au NPs, based either on a modification of the Turkevich method or on a reduction using sodium borohydride as reductor and thiols as capping agents. For the citrate-based method, we have used variations of the traditional Turkevich route as well as a “seeded-growth” method, introducing simplifications on the Piella et al. route (Piella et al. 2016). For the reduction with sodium borohydride, we used either cysteamine (C_2_H_7_NS (CYS)) (Niidome et al. 2004) or glutathione (C_10_H_17_N_3_O_6_S (GSH)) (Beato-Lopez et al. 2017; Beato-Lopez et al. 2012) as capping agents. Both capping agents contain a thiol group to be linked to the NP surface, as well as an amino terminal group (in the case of CYS) or both amino and carboxylic groups (in the case of GSH), that remain free to be cross-linked with other biomolecules, as we have previously shown (Beato-Lopez et al. 2017). Thus, starting from different synthetic routes, we obtain NPs with different capping, size, and homogeneity (Albanese et al. 2012; Chithrani et al. 2006; Li et al. 2018; Silvestri et al. 2017; Verma and Stellacci 2010).

We have analyzed size average, homogeneity, and structural characteristics of the obtained Au NPs. We have obtained four different capped gold NPs with average sizes ranging from 3 to 10 nm, which is important for biomedical applications, as many body barriers fall in the sub-10 nm range, while an increase in toxicity has been shown for NPs smaller than 2 nm (Khan et al. 2018; Lewinski et al. 2008; Pan et al. 2007; Sukhanova et al. 2018; Wozniak et al. 2017).

We have also studied NP surface characteristics and their colloidal behavior, as well as their stability at different pH values, particularly in the physiological pH range.

As the objective of these synthesis is to obtain functionalized gold NPs useful for biomedical applications, we have also studied their optical properties: optical absorption and photoluminescence, in order to analyze the interest of these NPs as bioimaging agents. We have studied the influence of NP size and capping on the LSPR band and on their PL emission, in order to evaluate NPs with potential for biomedical applications and analyzed the cytotoxicity of NPs, not only in transformed cell lines but also in primary cells, to closely mirror the situation in clinical settings.

Our purpose is to obtain biocompatible functionalized Au NPs and to study the influence of functionalization on their optical properties, stability, and cytotoxicity. Functionalization is a key and common factor for all biomedical applications, such as X-ray imaging and biosensing, and although the PL intensity of these Au NPs is not comparable to that of conventional biomarkers, their higher biocompatibility may facilitate their introduction in clinical settings where their emission intensity is enough and additionally PL characteristics can be an asset for the follow-up and complementation of any other application.

## Experimental

### Chemicals

Tetrachloroauric acid (HAuCl_4_), sodium citrate (Na_3_C_6_H_5_0_7_), tannic acid (C_76_H_52_O_46_), sodium borohydride (NaBH_4_), cysteamine (C_2_H_7_NS), and reduced glutathione (C_10_H_17_N_3_O_6_S) were purchased from Sigma-Aldrich and used as received. All the chemicals were of analytic grade, and Milli-Q water was used for all experiments.

### Au NPs preparation

Four different types of gold NPs were synthesized starting from the reduction of Au^3+^ cations present in the HAuCl_4_. Two different reducing agents were used: sodium citrate for synthesis A and B, and sodium borohydride for synthesis C and D.

#### Synthesis A: method based on the traditional Turkevich reaction

Briefly, 20 mL of a 0.01 M HAuCl_4_ aqueous solution was heated until 97 °C with vigorous stirring. Once this temperature was reached, 2 mL of a sodium citrate aqueous solution was added, in a HAuCl_4_/Na_3_C_6_H_5_0_7_ molar ratio of 1:5. After 10 min of strong stirring, the solution was stored at 8 °C in the protected from light.

**Fig. 1:**
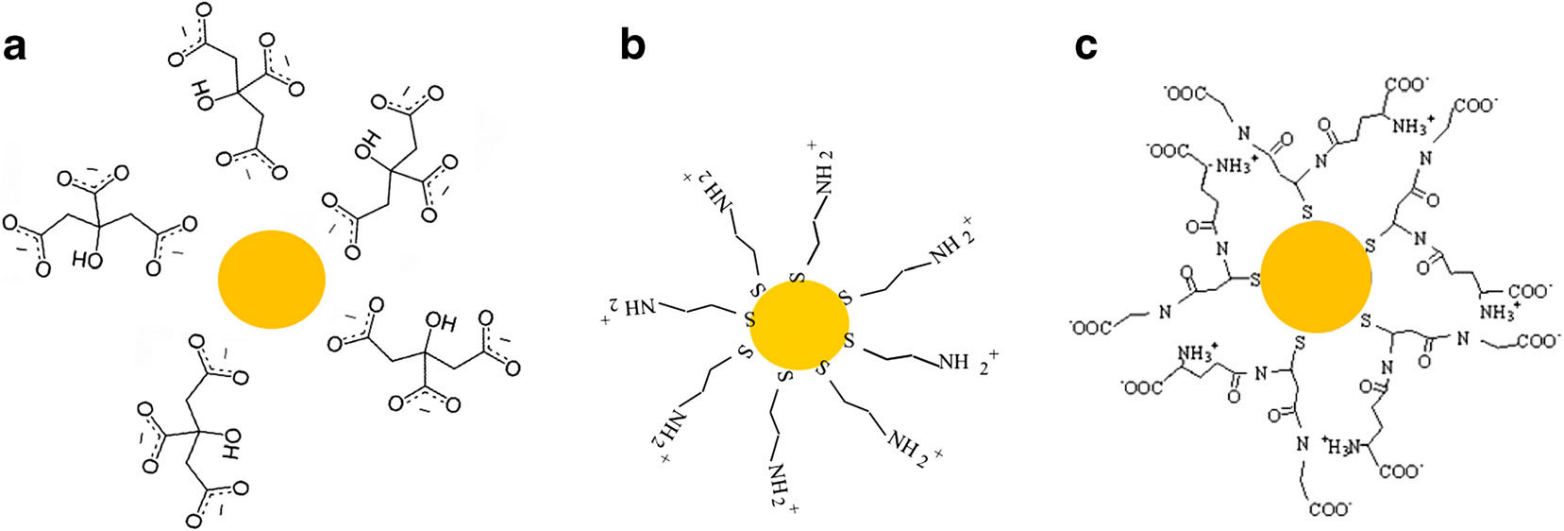
Schematic representation of different Au NP capping agents. **a** NP obtained by synthesis A or B (named Au-C and Au-TC, respectively): spherical gold core surrounded by citrate ligands. **b** NP obtained by synthesis C (named Au-CYS): the gold core can link to the thiol groups on the CYS molecule. **c** NP obtained by synthesis D (named Au-GSH): the gold core is bound to GSH thiol groups

A schematic representation of NPs obtained from this method is shown in Fig. 1a. We represent a spherical gold core surrounded by citrate ligands. Even if the precise structure of capped NP can be much more complex, this schematics allows us to have a graphical representation of the system. The code Au-C is used for this type of NPs.

#### Synthesis B: seeded-growth method

This synthesis is also based on the reduction of HAuCl_4_ using sodium citrate, but including the addition of a co-reducing agent, in order to promote the fast nucleation and successive growth in order to homogenize NP size. The process is based on that proposed by Piella et al. (Piella et al. 2016) but introducing some simplifications.

We have used a 1:10, HAuCl_4_/Na_3_C_6_H_5_0_7_ molar ratio. A co-reducing agent, the tannic acid, is used to promote gold nucleation. The reaction occurs in the following steps: In the first step, 1 mL of HAuCl_4_ 0.025 M was added to a three-necked round-button flask containing a mixture of 150 mL 2.2 mM sodium citrate and 0.1 mL 2.5 mM tannic acid, previously heated at 70 °C. In a few minutes, a brownish-orange color was observed, indicating formation of small gold nuclei. After 5 and 20 min, 55 mL of the solution was drawn from the flask and 0.5 mL 0.025 M HAuCl_4_ and 55 mL 2.2 mM citrate solution were added to the reaction. The reaction continued for 20 more minutes before the final solution was cooled and stored at 8 °C in the dark. These two last steps were carried out to complete the homogenous growing of the seeds formed in the initial step.

As in the case of synthesis A, the final gold core obtained by this process must be surrounded by citrate ligands, added in excess in the three steps of the reaction (see schematics in Fig. 1a). The code Au-TC is used for this type of NPs in order to distinguish them from the ones obtained with the same capping but from synthesis A.

#### Synthesis C: CYS capped Au NPs

The reaction is based in the reduction of HAuCl_4_ in aqueous media using NaBH_4_ as reducing agent and cysteamine (CYS) as capping agent to control the growth and to avoid aggregation (Niidome et al. 2004). We have modified previous methods to decrease and homogenize the average size of NPs.

Forty milliliters of a 0.01 M HAuCl_4_ aqueous solution was mixed with 1 mL of a 0.08 M CYS aqueous solution. The mixture was vigorously stirred for 30 min under a nitrogen atmosphere. Subsequently, an aqueous solution of NaBH_4_ was added dropwise, in a 1:0.5 HAuCl_4_/NaBH_4_ molar ratio. After 30 additional minutes, the red wine color solution was kept at 8 °C in the dark. A schematic representation of the final NP obtained by synthesis C is shown in Fig. 1b. The gold core can link to the thiol groups on the CYS molecule. The code Au-CYS is used for this type of NPs.

#### Synthesis D: GSH capped Au NPs

In this case, the reduction also takes place using NaBH_4_ in aqueous media. However, in this case, GSH was used as capping agent.

Briefly, 10 mL 0.01 M HAuCl_4_ aqueous solution was mixed with 10 mL 0.026 M GSH aqueous solution. The mixture was vigorously stirred for 30 min under a nitrogen atmosphere. A NaBH_4_ aqueous solution was added dropwise, in a 1:5 HAuCl_4_/NaBH_4_ molar ratio. After 30 additional minutes, the red wine color solution was stored at 8 °C protected from light. Based on our previous works (Beato-Lopez et al. 2017; Beato-Lopez et al. 2012), we have designed this rapid route to obtained small GSH capped gold NPs. Figure 1c shows a schematic representation of the final NP obtained by this synthesis. The gold core is bound to GSH thiol groups. The code Au-GSH is used for this type of NPs.

### Characterization

Structural studies as well as particle average size estimations have been obtained from transmission electron microscopy, using a FEI Nova NanoSEM 450, operating at STEM (scanning transmission electron microscopy) mode. Samples were prepared by depositing 10 μL of an Au NP colloidal solution that was drop-casted onto a holey-carbon-coated Cu grid and dried for 5 h. Images were obtained either in dark field (DF) or bright field (BF). High-resolution images have been obtained transmission electron microscopy (TEM) using a JEOL 2010.

Dynamic light scattering (DLS) was used to measure the hydrodynamic NP size. Measurements were carried out at 25 °C, using a Nanotrac Wave (Microtrac) equipment, with a 0.5 cm path cell. This equipment also allows for the *Z* potential to be measured which evaluates colloidal solution stability, as well as NP surface charge.

The study of gold nanoparticles functionalized with different capping agents was analyzed by Fourier transformed infrared spectrophotometry (FTIR). The spectra were recorded in the region 4000 to 400 cm^−1^ by means of a Bruker Tensor 37 spectrophotometer.

Au NP LSPR has been evaluated studying the UV-Vis absorbance of each NP colloidal solution. Absorption spectra were acquired with a Lambda 19 PerkinElmer spectrophotometer. Position and profile of LSPR band provide information about NP dispersion and allow for the estimation of NP average size.

Photoluminescence excitation and emission spectra have been recorded in a PTI Quantamaster fluorometer using a Xenon arc Lamp at 150 W and a computer controller QuadraScopic monochromator. Fluorescence lifetime measurements have been made by means of a LaserStrobe technique by using a nitrogen/dye laser GL-3300 providing 440 kW peak power at 5 Hz with a pulse width of 1 ns (1.68 mJ/pulse for N_2_). The N_2_ laser optical pulse and the timing electronics of LaserStrobe, which scans different time delays after pulse, allow for a temporal resolution of up to 100 ps.

### Cell culture

Primary cells were obtained from human peripheral blood samples from healthy donors upon signature of an informed consent and following approval by the Ethics Committee of the Puerta del Mar University Hospital, according to Spanish and European regulations. Peripheral blood mononuclear cells (PBMC) were isolated by density gradient centrifugation using lymphocyte separation media (Eurobio™, Montpellier, France). PBMC were stimulated with 1 μg/mL phytohemagglutinin-P (PHA) (Sigma™, Saint Louis, MO, USA) and cultured at 37 °C in a 5% CO_2_ atmosphere in Dulbecco’s modified Eagle’s medium (DMEM) containing 2 mM l-glutamine, 10 mM HEPES, 10% (*v*/*v*) heat-inactivated fetal bovine serum (FBS), 1% (*v*/*v*) non-essential amino acids (NEAA), 1% (*v*/*v*) sodium pyruvate, 50 μM 2-mercaptoethanol, 100 U/mL penicillin, and 100 μg/mL streptomycin (all from Life Technologies, Carlsbad, CA, USA). Forty units per milliliter IL-2 was added to the culture every 48 h for a total of 6 days before the experiment. CD3, CD4, and CD8 expression in PBMC blasts the day of the experiment ranged from 77.8 to 65% for CD3%, 65.3 to 49.3% for CD4, and 12.5 to 15.7% for CD8. Jurkat cells (American Type Culture Collection, Manassas, VA, USA) were cultured in the same conditions as PBMC, and CD3 expression was evaluated periodically and always found to be above 80% (in supplementary Fig. S.1, we show flow cytometry data of blood, PBMC, and Jurkat cells from a representative experiment).

### Cell viability, cytotoxicity, and proliferation assays

For both primary as well as Jurkat cells, 5 × 10^5^ cells were cultured in a 24-well plate, in the absence or presence of nanoparticles at 1.5 μg/mL or 15 μg/mL.

Cell viability was analyzed at 24 h after addition of NPs by an MTT-based assay as previously described (Vistica et al. 1991). Briefly, MTT reactant (Thiazolyl Blue Tetrazolium Bromide, TOX1-1KT, Sigma-Aldrich) was added to the cells in a 1:10 ratio (MTT solution/culture medium) and incubated during 2 h at 37 °C. Then, formazan crystals formed inside the cells were dissolved by adding MTT Solubilization Solution (M-8910, Sigma-Aldrich) at 1:2 with vigorous pipetting. Optical density at 570 nm was evaluated to quantify the amount of formazan crystals, which is proportional to the number of viable cells (background absorbance was measured at 690 nm and subtracted from the 570 nm measurement). Viability was compared to untreated controls (100%).

#### Cell cytotoxicity assay

Cells were cultured as for the viability assay and after 24 h, were harvested, and cell death was evaluated by staining with a live/dead fixable near-IR dead cell stain kit (Thermo Fisher Scientific™) and analyzed in a Cytoflex™ Flow cytometer (Beckman Coulter, Inc., Fullerton, CA).

#### Cell proliferation assay

To label dividing cells, 10 μM EdU was added to the cultures for 24 h in the presence or absence of the corresponding nanoparticles. Cells were subsequently harvested, fixed in the Click-iT™ fixative, and permeabilized with a saponin-based permeabilization and wash reagent (Click-iT™ EdU Pacific Blue™, Thermo Fisher Scientific™). Edu was bound to a Pacific blue dye by means of a click reaction for 30 min (Breinbauer and Kohn 2003), washed twice with a saponin-based permeabilization and wash reagent, and analyzed in a Cytoflex™ flow cytometer (Beckman Coulter).

### Optical microscopy

Jurkat cells 1 × 10^5^ per well in 200 μL (at 5 × 10^5^ cells/mL) were cultured without nanoparticles or in the presence of nanoparticles at 1.5 μg/mL or 15 μg/mL, subsequently washed with phosphate-buffered saline (PBS), and fixed in a solution containing 4% formaldehyde in PBS at room temperature, for 30 min. After an additional washing step, cells were centrifuged at 900 rpm for 5 min on adhesion poly-lysine slides, Polysine® VWR Collection by means of a Cytospin™ 4 Cytocentrifuge (ThermoFisher Scientific), washed and mounted with Fluoro-Gel mounting medium (Catalog #17985-10, Electron Microscopy Sciences). Slides were analyzed on an Olympus Confocal microscope. Cells were excited at *λ*405, and emission was collected at λ413–510 with a × 40 magnification. Images were analyzed with the Fiji distribution software (Schindelin et al. 2012) of ImageJ (Schneider et al. 2012). Percentage of blue (413–510*λ*)-emitting cells from total cell (from a transmission micrograph) were obtained from three independent experiments and analyzed by one-way ANOVA using the XLSTAT® Excell® plug in.

## Results and discussion

### Structural and chemical features

**Fig. 2:**
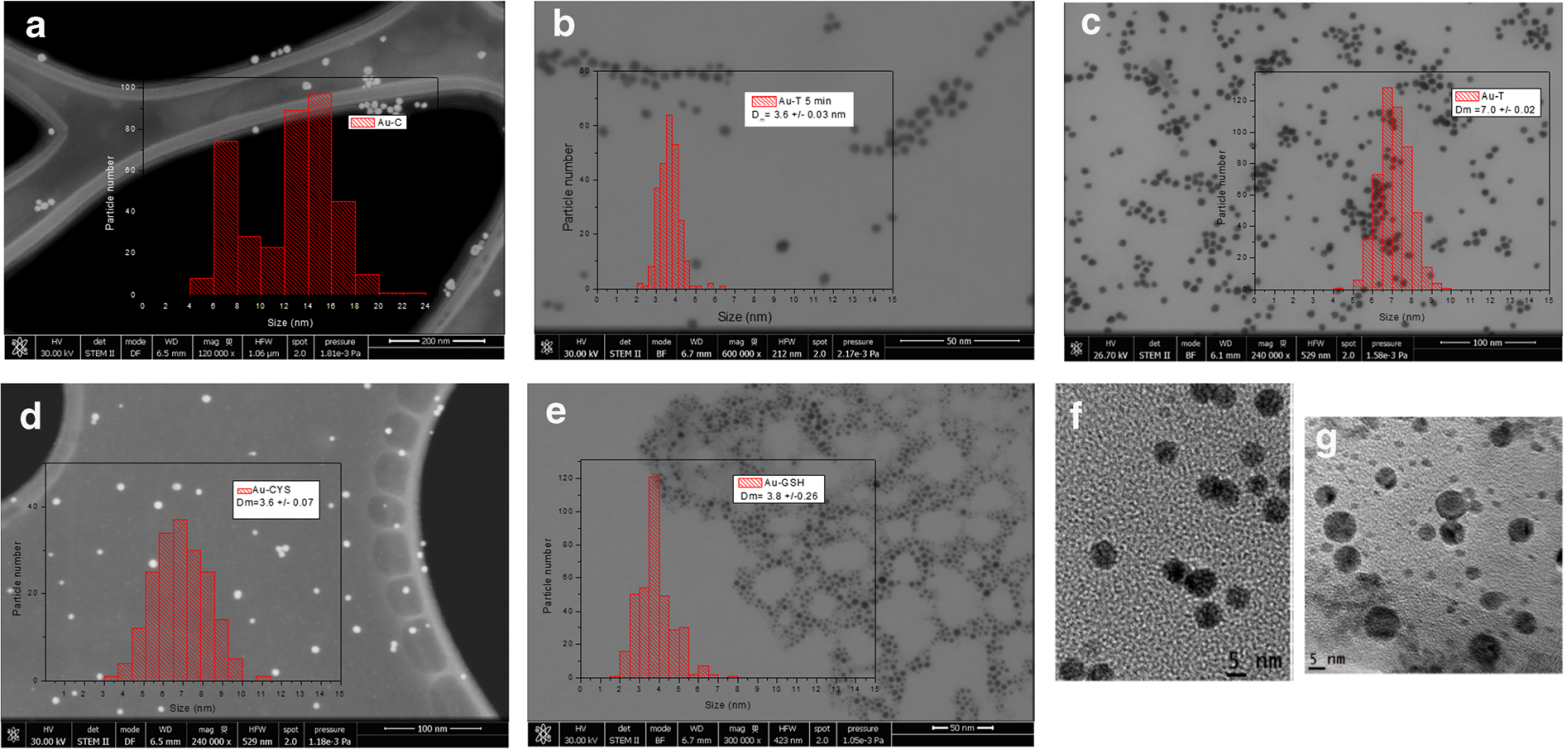
TEM micrographs obtained from different Au NP capping agents: **a** Au-C, **b** Au-TC after 5 min of synthesis, **c** Au-TC after 45 min of synthesis, **d** Au-CYS, e Au-GSH. Corresponding histograms displaying particle size distribution are shown in the right panels: **f** and **g** are high-resolution images for Au-TC and Au-CYS, respectively

TEM micrographs of the obtained gold NPs reveal the formation of relatively small and well-dispersed NPs with homogeneous size distributions and without aggregation (Fig. 2a–e show a general view for the different Au NPs). Insets show histograms for each type of NP, showing the size distribution obtained from the analysis of more than 300 NPs like those shown in micrographs. NP average sizes obtained from the fitting of these histograms to a Gaussian function are shown in Table 1. Au-C NPs prepared by synthesis A (classical sodium citrate synthesis) show a wider average size, with two main average sizes centered at 7.5 and 14.5 nm, respectively. Size distribution for these NPs shows a bimodal profile, indicating either the inhomogeneous growth or the aggregation of gold cores. Figure 2b shows the micrograph corresponding to Au-TC NPs after 5 min of synthesis (for this measurement, an aliquot of the colloidal solution was drawn 5 min after starting the reaction). We can observe formation of gold cores of an average size of 3.5 nm, with a homogeneous size distribution. The use of a strong co-reducing agent (tannic acid) promotes rapid nucleation and a consequent formation of small nuclei. At this point, the reaction is in the initial phase and the rate of nuclei production is faster than the rate of Ostwald ripening, leading to small NPs. After completion of the synthesis (45 min, Fig. 2c), Au-TC NPs reach an average size of 7.0 nm with a low size dispersion. As the reaction progresses, the rate of Ostwald ripening takes over and we have slightly bigger sizes. In this case, modifications included in the classical Turkevich route lead to homogenization of NP sizes. Rapid formation of gold nuclei in the first step of the reaction allows for a homogeneous growth yielding NPs with high monodispersity.

**Table 1:** TEM and DLS size estimates and values for *Z* potential for each NP type

NP code	TEM size (nm)	DLS size (nm)	*Z* potential (mV)
Au-C	14.5 ± 0.28	4.9 ± 0.1	− 158
Au-TC	7.0 ± 0.02	6.9 ± 0.4	− 99
Au-CYS	6.5 ± 0.07	8.0 ± 0.1	+ 88
Au-GSH	3.8 ± 0.26	3.6 ± 0.2	− 140
Au-TC 5 min	3.6 ± 0.03	3.5 ± 0.2	− 98

Thiol capped NPs (Fig. 2d, e) show average sizes of 6.5 nm and 3.5 nm for Au-CYS and Au-GSH, respectively (Table 1). Au-CYs NPs (Fig. 2d) show an average size of 6.5 nm, a diameter similar to that obtained for Au-TC. However, high-resolution images for these two types of NPs (Fig. 2f, g) show a more homogeneous distribution for Au-TC.

Au-GSH NPs (Fig. 2e) show a particularly small size distribution. We have previously reported production of small GSH capped NPs by means of a high GSH/Au molar ratio. The average size of this type of NPs is similar to that of Au-TC after a 5-min reaction (Fig. 2b); however, size distribution for Au-GSH NPs is considerably wider. Indeed, even though the micrograph corresponding to Au-GSH (Fig. 2e) shows a majority of small NP, NPs with sizes higher than the average value are also present.

Summarizing these results, we have obtained Au NPs with average sizes from 3.5 to 7 nm. When we use the seeded-growth method, we obtained slightly higher monodispersity than when we directly capped using thiol ligands. However, thiol capped Au NPs have the advantage of being directly functionalized for biomedical applications.

In order to compare TEM diameters with hydrodynamic sizes for NPs in solution, DLS experiments were performed. Average sizes obtained from these measurements are shown in Table 1. The general good agreement between TEM and DLS diameters indicates the absence of NP aggregation in the water colloid, and consequently, a well-established Au NP colloidal systems. However, DLS sizes should show the effect of capping on the hydrodynamic sphere. Interestingly, we only find this effect in Au-CYS. DLS size distribution for Au-CYS (supplementary Fig. S.2.a) shows an average size of 8 nm, slightly bigger than the TEM diameter for this NP. In this case, the differences between DLS and TEM diameters could be due to the effect of CYS capping on the hydrodynamic diameter. Figure S.2.b in the supplementary material shows the DLS distribution for Au-TC NPs. In this case, there are no significative differences between DLS and TEM average sizes. However, comparing TEM and DLS distribution for this sample (Figs. 2c and S.2.b), we can appreciate that DLS distribution shows a higher portion of NPs with sizes bigger than the maximum, which could mask the influence of capping on the hydrodynamic diameter. DLS distribution is slightly wider than TEM distributions (with a higher size dispersion) which can explain the influence of capping on hydrodynamic size. We obtained similar results for Au-GSH NPs. Other authors also find this correlation between TEM and DLS sizes, indicating that we work at the detection limit for the DLS instrument and thus small NPs with very low scattering are less detected. In the case of Au-C NPs, DLS sizes are considerably smaller than the corresponding TEM sizes, probably due to a higher size dispersion, that cause a lower precision in the average values obtained from both methods.

In all cases, we obtained Au NPs with high stability in aqueous colloidal solutions.

*Z* potential values obtained for each type of Au NP as prepared are also shown in Table 1. *Z* potential values are high for all NPs, pointing out to their high stability in water colloidal solution and, consequently, to their suitable features for biomedical applications. These measurements shed information on the NP surface charge sign that can be relevant for subsequent cross-linking applications. All NPs show negative values for *Z* potential, except Au-CYS NPs, probably due to the protonation of its amino terminal groups (see Fig. 1b).

**Fig. 3:**
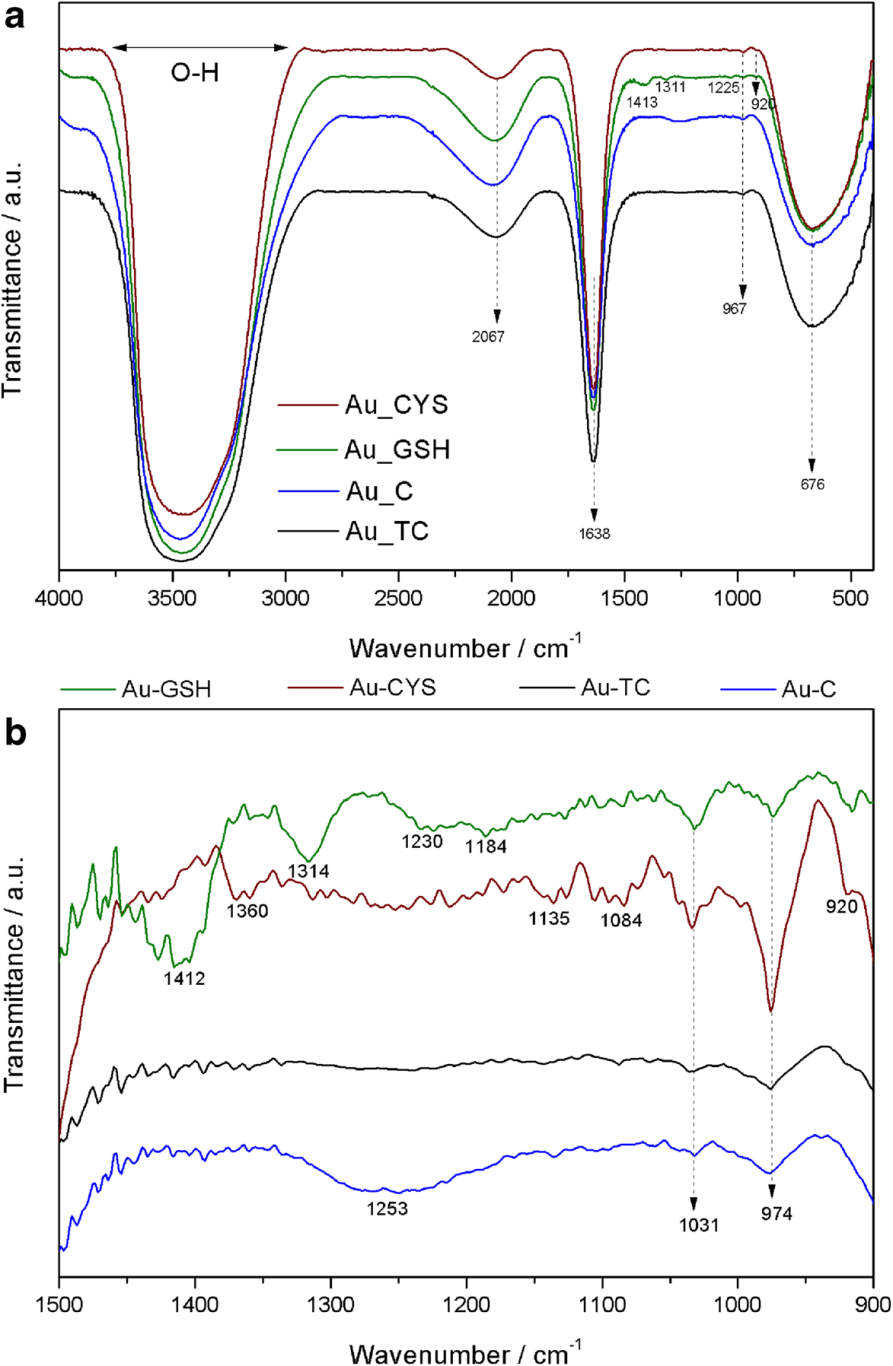
**a** FTIR spectra for the different Au NPs. **b** Amplification of the region between wavenumber 1500 and 900 cm^−1^

The functional groups in the samples were analyzed using FT-IR measurements, with the objective to analyze differences between Au NPs capped with different ligands. Figure 3a shows FT-IR spectra obtained for the four types of Au NPs. All NPs show similar spectra with the main bands in the same positions. The broadest peak around 3467 cm^−1^ can be attributed to stretching O-H vibration, and the band at 1638 cm^−1^ is frequently attributed to C-H stretch. All the spectra show a peak at 675 cm^−1^ corroborating the presence of Au NPs. When we amplify the region between 1500 and 900 cm^−1^ (Fig. 3b), we can appreciate some differences. As expected, Au-C and Au-TC, NPs with the same chemical surround, show the same profile, with no significate differences in the peak positions. Au-GSH and Au-CYS show a peak at 1030 cm^−1^ that could be assigned to C-S stretching vibrations. Au-GSH shows two more peaks, one at 1412 cm^−1^, assigned to asymmetric stretching vibration of carboxylate groups (COO–), and another at 1314 cm^−1^, associated with stretching vibration of the C-N of amide. Finally, in the case of Au-CYS, the peak at 1360 cm^−1^ can be associated with C-N stretching coupled with N-H deformation (Anand et al. 2017; Mathialagan and Shanmugam 2018).

### Optical behavior

#### UV-visible absorption

**Fig. 4:**
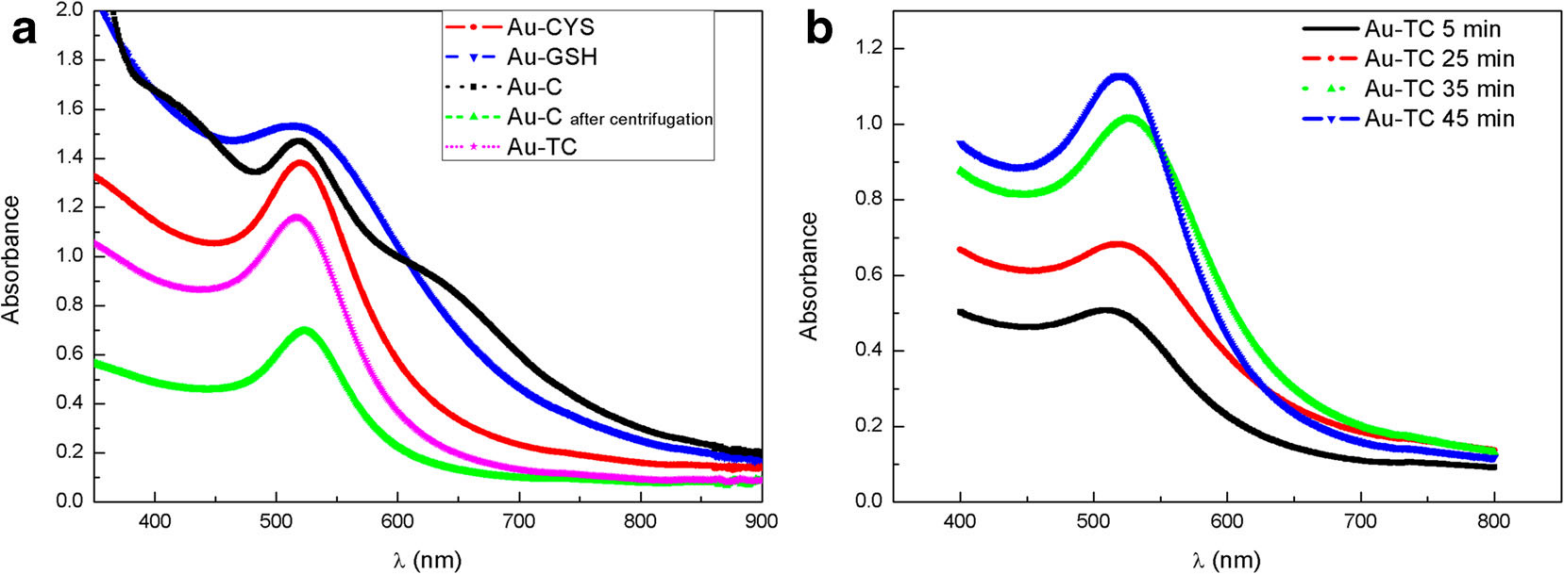
UV-visible spectra for the four different Au NP solutions (**a**) and for Au-TC NPs during the growing process (**b**)

The presence of gold NPs in the obtained colloidal solutions has also been confirmed by UV-visible absorption. Position and width of LSPR bands give information about NP size and homogeneity. Figure 4a shows the UV-Vis absorption spectra for the different Au NP colloids. Wavelength values for LSPR bands are shown in Table 2. All NPs show the characteristic LSPR absorption band around 520 nm, indicating the presence of small gold NPs. No significant differences are found for the position of the main absorption peak for different Au NPs. In the case of Au-C, a wide shoulder around 650 nm, together with a general increase in absorption intensity in the range of 600–800 nm, indicates the presence of bigger or non-spherical NPs. Interestingly, after centrifugation, this shoulder disappears, indicating that bigger NPs are eliminated with the centrifugation process.

**Table 2:** LSPR wavelength, absorbance values at 450 nm and at LSPR wavelength, and UV-Vis size estimates for different Au NPs

NP code	LSPR band position (nm)	*A*_LSPR_	*A*_450_	UV-Vis size (nm)	TEM size (nm)	*N* (NP number per cm^3^)
Au-C	520	0.86	0.60	9.2	14.5 ± 0.28	1.63 × 10^15^
Au-TC	520	1.13	0.88	4.3	7.0 ± 0.02	1.25 × 10^15^
Au-CYS	520	1.38	1.05	4.7	6.5 ± 0.07	1.80 × 10^15^
Au-GSH	518	1.54	1.49	1.7	3.8 ± 0.26	3.72 × 10^15^
Au-TC 5 min	508	0.51	0.46	2.5	3.6 ± 0.03	1.50 × 10^15^

The Au-GSH LSPR band shows a wider profile than that of the other colloids, which can be a consequence of the higher size dispersion in small NPs. The high number of surface atoms in small NPs, increases the damping of oscillating electrons at conduction bands. This effect can be even higher for small thiol capped NPs, due to the loss of itinerancy of electrons involve in Au-S bonding (Crespo et al. 2004; Fernandez et al. 2006).

NP diameters calculated from UV-Vis absorption spectra are also shown in Table 2. Haiss et al. proposed a method to estimate both NP size and particle number density, from absorption spectra for NPs smaller than 30 nm (Haiss et al. 2007). This model takes into account the LSPR absorbance in relation to the typical Au absorbance at 450 nm. They stablish a dependency between the logarithm of NP size and the ratio between the absorbance at the LSPR absorbance (*A*_LSPR_) and the absorbance at 450 nm (*A*_450_). Haiss et al. (2007) use the following expression to estimate NP size from UV-Vis spectra:

$$ d=\exp \left({B}_1\frac{A_{SPR}}{A_{450}}-{B}_2\right) $$

where *B*_1_ (3.55) and *B*_2_ (3.11) are the inverse of slope and intercept, respectively, obtained from the linear fit between the representation of the logarithm of NP size versus ratio *A*_SPR_/*A*_450_.

Table 2 shows absorbance values at 450 nm and at the LSPR wavelengths for each NP size, as well as the UV-Vis sizes obtained from the Haiss method (Haiss et al. 2007). The TEM sizes have been included in order to facilitate comparison.

UV-Vis sizes obtained using the above equation (Table 2) are in general smaller than those obtained from TEM; however, they show the same general tendency. This UV-Vis method to calculate NP diameters, based in the difference between Au absorbance at 450 nm and LSPR Au band, considers only the smaller NPs that contribute to the main LSPR maximum band. In the case of TEM estimations, we also count bigger NPs, absorbing at higher wavelengths. Although both methods give the smallest diameter values for Au-GSH NPs, the values obtained from the UV-Vis method for the Au-GSH NPs are particularly small. When we study the micrographs obtained from TEM for this NPs (Fig. 2e), we can observe some NPs bigger than 3.5 nm, which increases the average size. This proportion of bigger NPs is not included in the UV-Vis size estimation, according to the Haiss model.

These authors also propose a method to estimate the number density of NP. They propose analytical correlations between extinction efficiency and NP size, allowing an estimation of particle concentration (Haiss et al. 2007; Hendel et al. 2014; Tang et al. 2018). The values obtained from this estimation are also shown in Table 2.

Figure 4b shows the temporal evolution of the UV-Vis absorption spectra for Au-TC NPs. At different time intervals during the synthesis (5, 25, 35, and 45 min), aliquots of the colloidal solution were drawn and cooled, and the UV-Visible spectra measured. The LSPR band shifts from 508 nm after 5 min of reaction, to 520 nm at the end of the synthesis (45 min). As we have evaluated by TEM, small Au-TC nuclei are formed after 5 min of synthesis (Fig. 2b). Absorbance values at 450 nm and at the LSPR band have a relation with size and number of NPs in suspension. So, the LSPR for Au-TC at 5 min reaction is wider and centered at 508 nm as we have small NPs, but as the reaction progresses, the number of nuclei saturates and they grow due to Ostwald ripening, leading to a narrower band at 520 nm. The interest of this method resides in the fact that small NPs (nuclei obtained at the first step of reaction) with high size homogeneity and capped with citrate molecules can be obtained. When we compare size values obtained from TEM and UV-Vis (Table 1), we can observe a very good agreement between both methods, pointing out to a higher size homogeneity for Au-TC after 5 min. Au-GSH and Au-TC after 5 min show a similar TEM size. However, the UV-Vis size is considerably smaller for Au-GSH NPs due to the presence of bigger NPs that are not taken into account in the UV-Vis estimations. Although Au-GSH and Au-TC at 5 min have a very similar size average, the size homogeneity is considerably higher in the case of Au-TC. This seeded-growth method allows for the production of small NPs capped with citrate molecules, with a similar size than those obtained using GSH as capping agent.

**Fig. 5:**
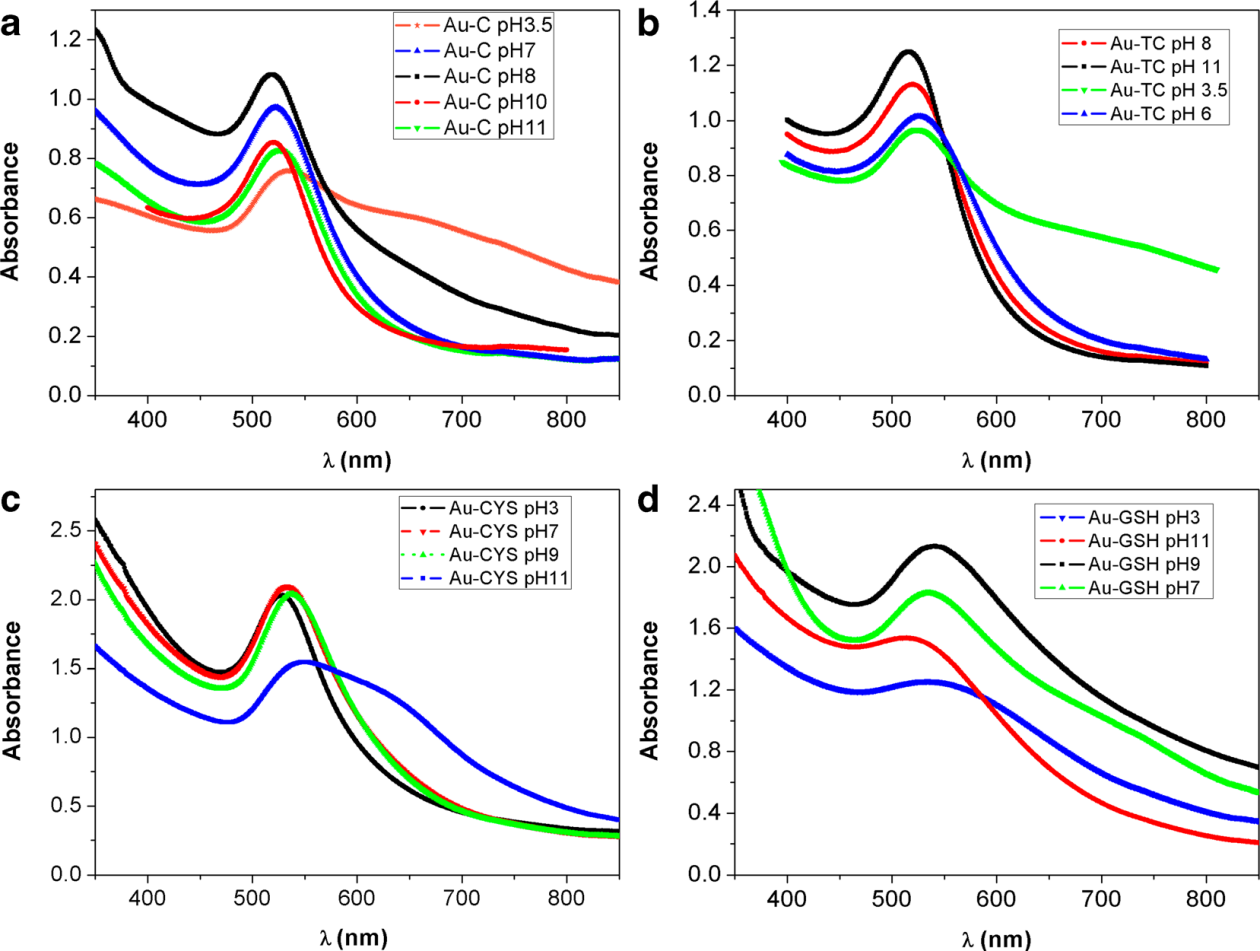
Absorption spectra at different pH values for Au-C (**a**), Au-TC (**b**), Au-CYS (**c**), and Au-GSH (**d**) colloidal NP solutions

In order to evaluate the pH range in which these functionalized NPs are stable, so we can select appropriate applications for each type of NP, we have studied the UV-Vis absorption at different pH values. Figure 5 shows UV-Vis absorption for the four different types of NPs in colloidal solutions at different pHs.

NPs capped with citrate molecules, Au-C and Au-TC, show a very good stability in the 6 to 11 pH range. In this case, for acid pH, the LSPR band becomes wider and the absorption in the 600–800 nm range increases, indicating aggregation of NPs. In the case of Au-CYS NPs, the absorption spectrum shows a narrow LSPR band at acid pH values, keeping a similar profile until pH 11 is reached, after which, the spectrum starts to show evidences of Np aggregation. In the case of Au-GSH NPs, the absorbance spectrum shows a well-defined LSPR band in neutral and basic solutions, which slightly broadens at pH 3, indicating the high stability of this type of NPs in a wide range of pH. Table S.1 in supplementary material shows *Z* potential values obtained for NPs at different pH. Summarizing, citrate capped NPs are more stable at neutral and slightly basic pH, whereas the pH range of stability is widened for thiol capped NPs, mostly for Au-CYS NPs which show a high stability in acidic media. Interestingly, all NPs show relatively high stability at physiological pH values making them suitable for biomedical applications.

#### PL emission

**Fig. 6:**
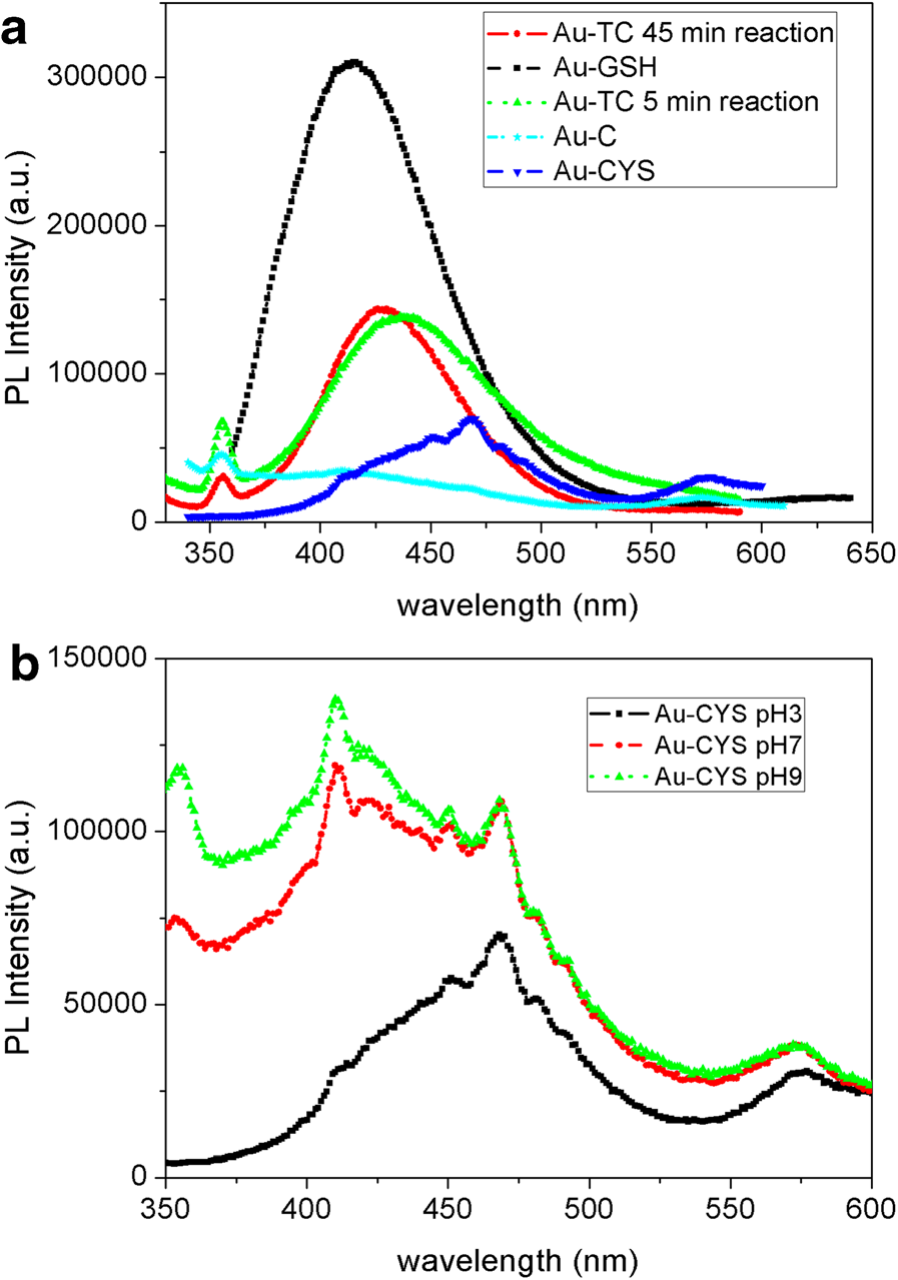
PL emission spectra for Au NP solutions excited at 317 nm (**a**). PL emission spectra for Au-CYS NP colloidal solutions at different pH values (**b**)

Photoluminescent behavior of Au NPs can give information about the structure and influence of the ligands on metal cores. Moreover, this is an interesting property for the use of Au NPs as biomarkers. Figure 6a shows PL emission spectra recorded for the different Au NP colloidal solutions, exciting at 317 nm. This excitation wavelength has been selected on the basis of the excitation spectra.

Samples Au-GSH, Au-TC, and Au-TC after a 5-min reaction show relatively strong emission bands in the blue range of the spectra. The emission is considerably lower for Au-CYS and Au-C. As we have previously mentioned, the origin of photoluminescence in gold NPs is still not clear and the emission band can be attributed to the interband transition Au 5d^10^ to 6sp and also to the capping at the ligand-metal charge transfer transition. It has been previously reported that if the PL is due to the transitions in the metal core, the emission wavelength could be tunable, by increasing NP size, due to the quantum confinement effect (Chen et al. 2009). In our study, samples Au-TC (45 min reaction) and Au-TC after 5-min reaction have exactly the same capping ligand, being the average size, the only difference between them. However, the maximum wavelength for the emission bands are 428 nm for NPs with higher diameter (Au-TC) and 435 nm for NPs with lower diameter (Au-TC after 5 min). There is no clear correlation between size and wavelength emission. Moreover, if we compare the emission of NPs with similar average size but different capping, samples Au-TC after a 5 min reaction and Au-GSH, we find that they show emission maxima at 435 and 416 nm, respectively. This behavior seems to indicate that PL emission in these NPs is not mainly determined by the metal core transitions, suggesting the influence of capping-core transferences. However, Au-C NPs also capped with citric acid show a very weak emission. Au-C NPs have a considerably bigger size dispersion than Au-TC NPs. Although both types of NPs are capped with citrate ligand, the capping is much more effective in the case of Au-TC, in which, due to the high size homogeneity, approximately the same number of citrate molecules surrounds the gold core in all NPs, enhancing the intensity of PL emission. This capping is still more efficient in the case of NPs linked to GSH molecules (Au-GSH NPs) due to the strong donor character of the thiol group present in GSH molecules. The PL emission in the case of Au-CYS NPs is considerably weaker than that of Au-GSH, in spite of the thiol group also linked to the core surface. Considering the small average size of Au-GSH NPs, and our previous results demonstrating that a high proportion of GSH molecules are linked to NP cores of approximately 3-nm diameter, we hypothesize a strong influence of the thiol capping in these NPs. Au-CYS NPs have a higher average size and consequently less ligand-core transference contributing to the PL. We also have to consider that we have a smaller number of NPs per volume in the case of Au-CYS (Table 2). Moreover, their poor emission can also be a consequence of the influence of pH. The colloidal solution resulting from this synthesis has an acid pH (Au-CYS colloid has acid pH as prepared). Sun’s work demonstrated that the Au^3+^ has a higher tendency to be reduced at basic pH (Sun et al. 2011). Moreover, Liu et al. (Liu et al. 2014) find considerably higher QY values for gold NPs synthetized at pH 9. Figure 6b shows the PL emission spectra obtained for Au-CYS as prepared (pH 3) and at different pH values. The emission increases from acid to basic pH values, reaching the maximum intensity at pH 9. As pH changes from 3 to 6, a first peak around 416 nm appears. The gold nanoparticles are more stable in neutral and basic pH range. Even though the Au NPs are functionalized with CYS, when moving towards acidic medium, the increasing number of protons in solution can affect the Au-S bond, which can eventually lead to aggregation and a size increase for gold nanoparticles. The noisy emission band profiles suggest the presence of surface processes contributing to the fluorescence. The surface defects originated by the acid synthesis can lead to non-radiative processes, decreasing the PL emission of these NPs, in spite of the influence of the strong donor ligand. All these evidences, together with the high stoke’s shift (more than 100 nm), suggest a strong influence of NP surface in its PL behavior, mainly governed by ligand-metal transference transitions and by surface processes (Chen et al. 2009; Liu et al. 2014).

Results obtained from PL dynamic experiments for the NPs with higher emissions (Au-GSH and Au-TC) reveal the existence of two processes contributing to the fluorescence, since PL decays can be fitted to a biexponential curve. Table 3 shows PL lifetime constants, *τ*_1_ and *τ*_2_, as well as pre-exponential parameters, *A*_1_ and *A*_2_, obtained from the fitting. In all cases, there is a process characterized by the lifetime constant *τ*_1_ ranging between 12.7 and 17.6 ns, and a second process slightly faster than the first one, with a life constant, *τ*_2_, around 3 ns (Table 3). The low QY obtained for many authors in metal NPs has the origin in the existence of non-radiative processes. The fastest process found for our NPs, probably related to surface and mainly non-radiative transitions, has in all cases a higher weight than the slower one, which is probably associated with ligand-metal radiative transferences.

**Table 3:** Photoluminescence lifetime constants and QY values for Au-TC and Au-GSH NPs

Sample	*τ*_1_ (ns)	*τ*_2_ (ns)	*A*_1_	*A*_2_	QY (%)
Au-TC	15.5	3.5	16	284	0.9
Au-TC 5 min	17.6	3.0	153	2279	1.2
Au-GSH	12.7	2.6	23.5	1213	2.1

We have estimated QY of the NPs with higher emissions, Au-GSH and Au-TC, using quinine sulfate (56%) as standard (Table 3). The obtained QY values are relatively high compared with values traditionally find for gold NPs. The capping effect, and consequently the charge transfer between electron donor ligand and metal core in these NPs, enhances the radiative processes increasing the QY, an indication that these two types of Au NPs are candidates for imaging studies.

### Intracellular staining

**Fig. 7:**
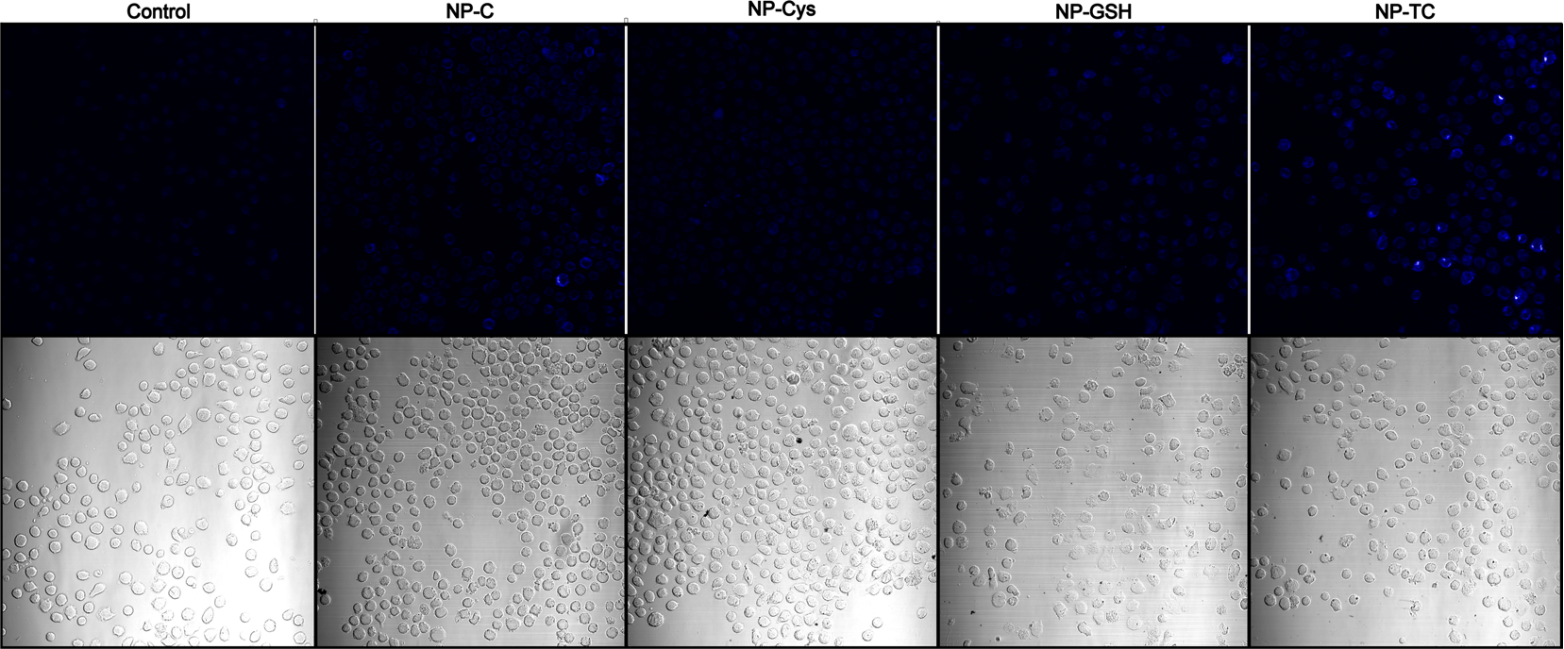
Fluorescence microscopy images of cells that were incubated with different NPs (Au-C, Au-Cys, Au-GSH, or Au-TC) or cultured in the absence of NPs as a control. The corresponding transmitted light microscopy images are shown in the lower panel. Images from three different experiments were analyzed and both total and positive cells were counted with the aid of FIJI® software. The percentage of fluorescent cells from three different experiments was analyzed for statistical significance by ANOVA (only NP-TC shows a statistically significant increase in fluorescence above control cells [*p* < 0.01])

We wanted to analyze whether the blue emission observed in Fig. 6 could be detected in cells by means of fluorescence microscopy, an application broadly used in Biomedicine. As shown in Fig. 7, cells cultured in the presence of NP-C, NP-GSH and NP-TC, have an increased blue fluorescence when compared to cells cultured in the absence of NP, although such difference is only statistically significant (*p* < 0.01) for NP-TC (Fig. 7). This results are in agreement with the highest PL emission observed for NP-TC nanoparticles and indicate that they not only emit blue fluorescence but can also enter cells. On the other hand, NP-CYS cultured cells showed a fluorescence higher than the one granted by its PL emission, probably due to the fact that their positive charged surface favors cellular uptake (Goodman et al. 2004); conversely, NP-GSH cultured cells show a lower emission than expected by the PL studies, probably due to the increased toxicity that selects cells that have incorporated less NPs. Uptake of particles of the size prepared in our experiments relies on active energy-dependent mechanisms that include dynamin-dependent (both clathrin and caveolin) mediated endocytosis (Ding et al. 2018), while larger or aggregated particles may mainly go through, by macropinocytosis (Gao et al. 2005). As the size and composition of the core Np are fairly similar, the differences seen in our experiments between NP very likely stem from differences in the corona (Cheng et al. 2015; Ding et al. 2018).

### Biocompatibility of NPs

#### Effect of NPs on cell viability

In order to analyze biocompatibility of the QD solutions developed, we analyzed their effect on cell toxicity, not only in a tumor cell line such as Jurkat, frequently used as a model for lymphocytes, but also directly in primary cells that more closely mimic the real situation in which NPs will eventually be used in clinical settings. Analysis in primary cells is relevant as tumor cells frequently have different sensitivities to lysis than their primary counterparts. As primary cells, we have chosen PHA blasts from PBMC as a representation of cells present in the blood. When extracted, they are composed mainly of lymphocytes (both T and B cells) as well as monocytes. The one used in the toxicity experiments was activated for 6 days (with a mitogen that resembles antigen stimulation), causing them to physiologically proliferate (or “blast”). This blasts react more readily to stimuli or toxic substances than their resting counterparts and thus, are more suitable for cytotoxic studies. The mitogen used activates only T cells; thus, after 6 days, only T cells remain (see staining in supplementary Fig. S.1). JKT cells have been selected, as it is a tumor cell line widely used as a T cell model for signal transduction and toxicity experiments; this way, we could compare primary cells that are proliferating physiologically vs tumor cells that are proliferating because they are tumors.

**Fig. 8:**
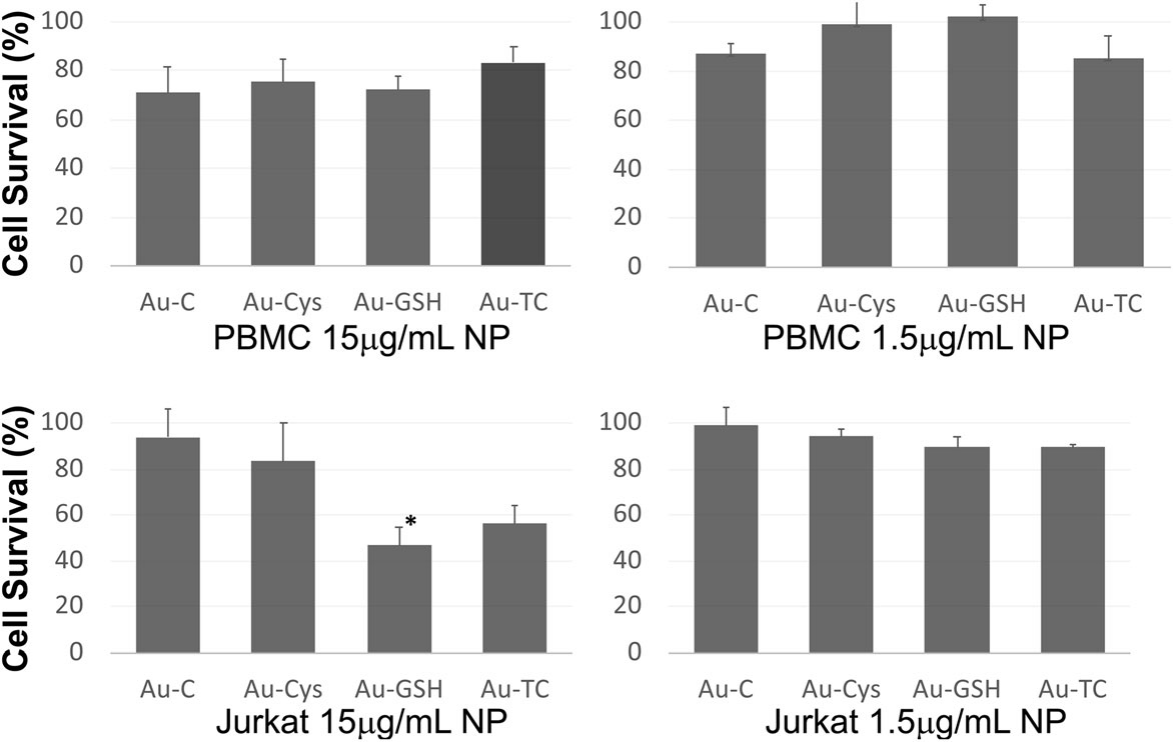
MTT cell viability assay of primary lymphocytes (upper panels) or Jurkat tumor cells (lower panels), incubated with the indicated NPs at 15 μg/mL (left panels) or 1.5 μg/mL (right panels). Results are represented as the mean ± standard deviation of at least three independent experiments performed in triplicate. Statistical analysis was performed using the GraphPad Prism 5.00 software. Significance was determined using a *t* test. Statistical significance is indicated by an asterisk (*p < 0.05)

We analyzed cell viability by MTT assay, as shown in Fig. 8; primary cells cultured in the presence of NPs at a very high concentration (15 μg/mL) showed a cell viability from 70 to 80% (71 ± 11%, 75 ± 9%, 72 ± 6%, and 83 ± 7% for Au-C, Au-CYS, Au-GSH, and Au-TC, respectively), while with the concentrations commonly used for this type of experiments in the literature (1.5 μg/mL), cell viability was close to 100% (87 ± 4%, 99 ± 15%, 102 ± 5%, and 85 ± 10% for Au-C, Au-CYS, Au-GSH, and Au-TC, respectively). Interestingly, when Jurkat tumor cells were analyzed, there is a significant effect of Au-GSH in cell viability (only 49 ± 5% of viable cells) and some effect for Au-TC (52 ± 9%), while the other NPs show viability profiles similar to that of primary cells (89 ± 10 and 72 ± 5 for Au-C and Au-CYS, respectively); again with NPs at 1.5 μg/mL, cell viability was close to 100% (99 ± 8%, 94 ± 3%, 89 ± 4%, and 89 ± 1% for Au-C, Au-CYS, Au-GSH, and Au-TC, respectively).

#### Effect of NPs on cytotoxicity

As the MTT assay measures the number of viable cells (those capable of transforming the MTT substrate) and such number depends not only on cells that have not died (and thus indirectly measures cytotoxicity) but also depends on proliferation, we used a state-of-the-art method to directly evaluate cell death, using flow cytometry, that directly gives a statistical evaluation of cell populations using a fluorescent dye that specifically stain dead cells, with excitation and emission features that are completely different from those of the NPs tested, thus preventing any overlapping in the detection.

**Fig. 9:**
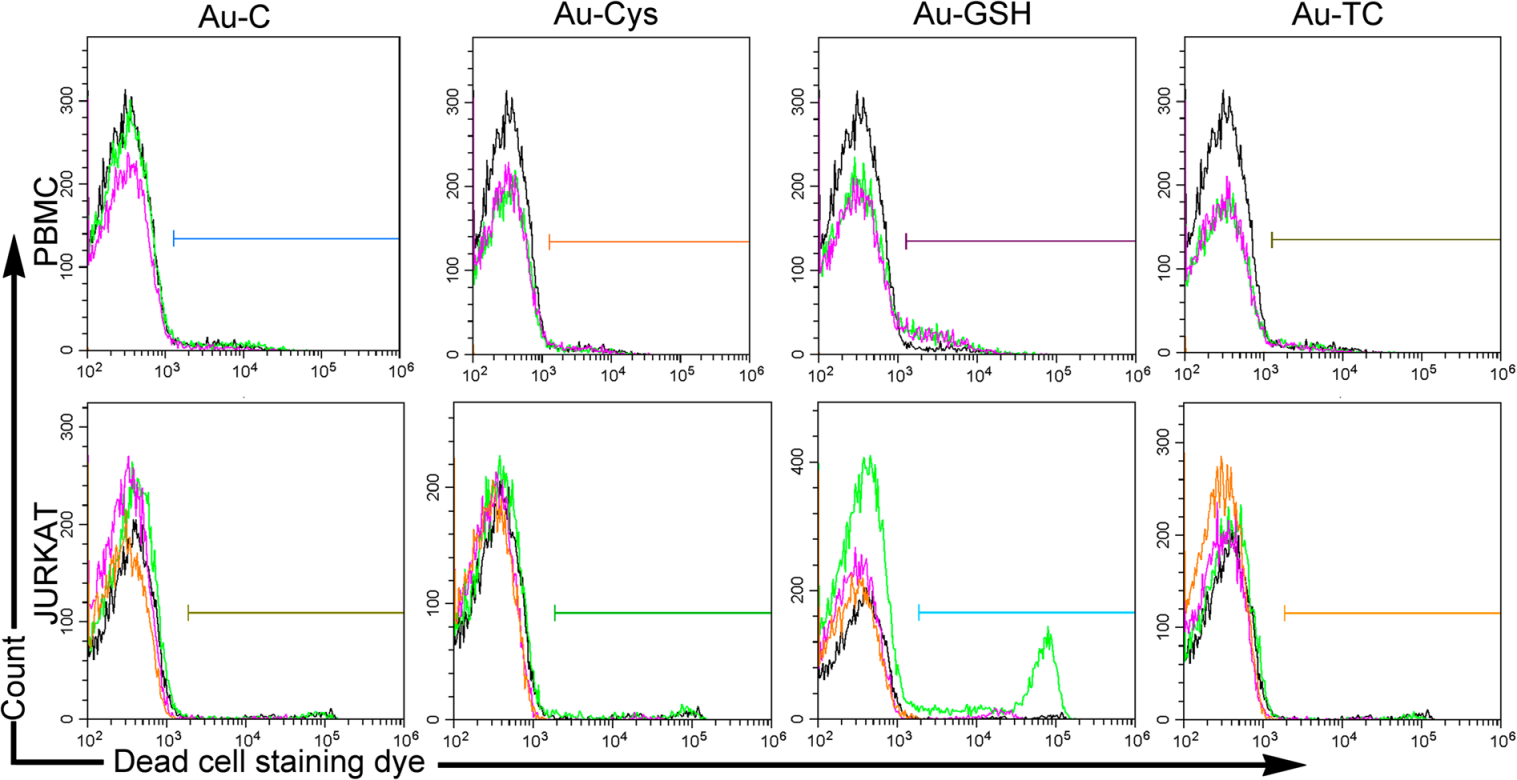
Flow cytometry dot plot showing the cytotoxicity profile of primary lymphocytes—PBMC—(upper panels) or Jurkat tumor cells (lower panels), cultured with the indicated NPs. Cells (at least 10,000) were electronically gated according to their size/granularity distribution. Histograms corresponding to three Live/dead™ cell staining (dead cells) experiments are shown for each Np. Within each histogram, different experiments for each NP are shown in different colors, with the control sample with no NP shown in black. According to a one-way ANOVA analysis (XLSTAT® Excell®), only PBMC cultured in the presence of Au-GSH showed a significant increase in cell dead when compared to cells cultured in the absence of NPs (*p* < 0.002)

As shown in the upper panel of Fig. 9, the percentage of dead primary lymphocytes (PBMC) in the presence of 15 μg/mL NPs was low for AU-C (2.46%), Au-CYS (2.35%), and Au-TC (2.63%) and similar to that of control cells in the absence of NP (2.27%), while it doubled for cells cultured in the presence of Au-GSH (4.65%). Jurkat cells (Fig. 9, lower panels) also showed an increased toxicity in the presence of Au-GSH, but while ANOVA analysis showed that it was significant for PBMC (*p* < 0,002), it failed to do so for Jurkat cells, probably due to a higher variability in the results (no toxicity was observed at 1.5 μg/mL). The difference observed between the number of viable cells in the MTT assay and the flow cytometry results stems partly from the fact that in the flow cytometry experiments, cells had been previously gated to select for the lymphocyte population and thus we are quantifying dead cells among a population that has not yet lost their size and granularity and has thus only recently initiated the dead path.

#### Effect of NPs on cell proliferation

**Fig. 10:**
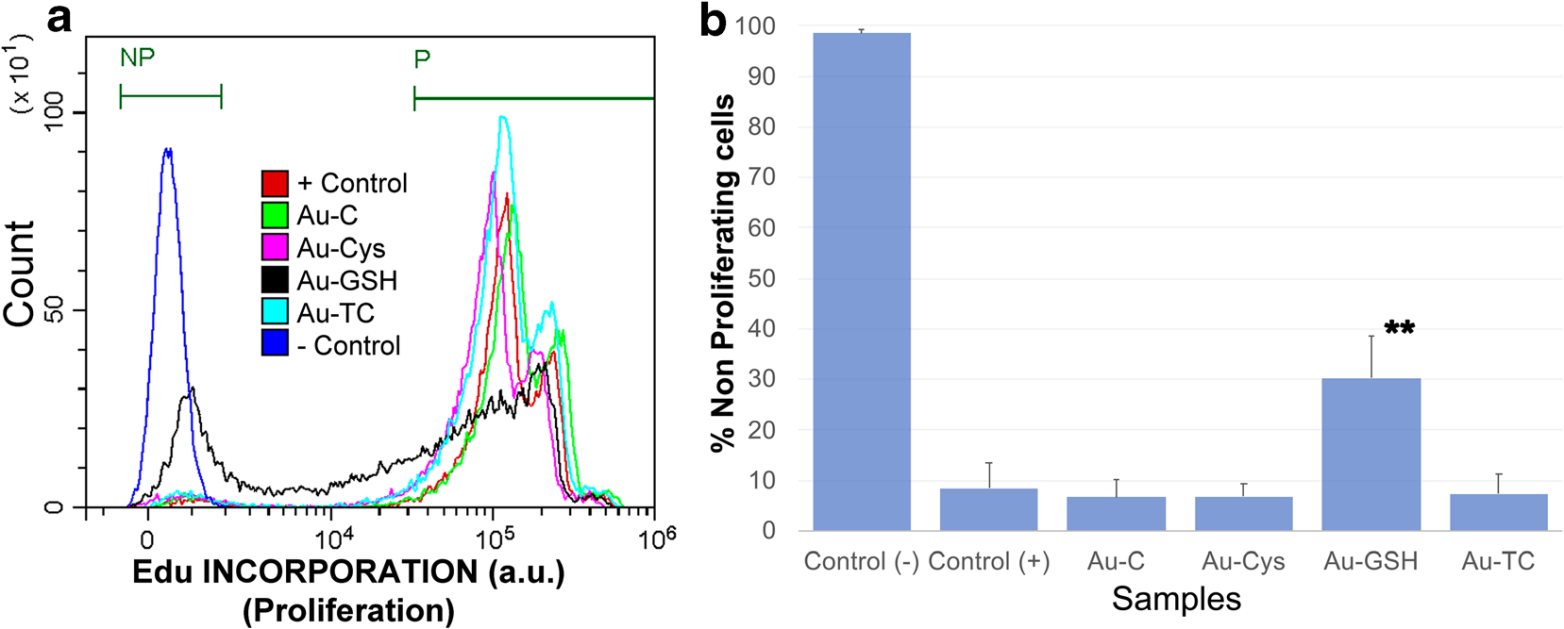
Detection of proliferating cells by EdU incorporation and labeling. Jurkat tumor cells were cultured with 10 μM EdU, in the presence or absence of the indicated nanoparticles at 15 μg/mL. Proliferation was determined as a function of Edu incorporation into cells’ DNA, detected by means of a Pacific blue fluorescent dye. EdU+ proliferating (gated in P) and non-proliferating cells (NP) were clearly and distinctly separated by FACS. Jurkat cells in the absence of NP were used as a positive control (+ control) for proliferation, as they are a tumor cell line that spontaneously proliferate in culture. Cells that have not incorporated Edu were used as negative control (− control). **a** Overlay histogram showing the proliferation profile of all samples. Samples had been electronically gated according to their size/granularity distribution. Individual samples as well as the gating strategy are shown in supplementary Fig. S.3. An overlay histogram of the ungated populations as well as results corresponding to individual ungated samples is shown in Fig. S.4.b) Average and standard errors for the percentage of non-proliferating cells under each condition (**p < 0.001)

As there was still some discrepancy between the viability and cytotoxicity assays, we wanted to determine whether a differential effect on proliferation could be responsible for the difference seen for the Au-GSH cultured Jurkat tumor cells. The most accurate method to analyze proliferation is by directly measuring DNA synthesis. Thus, we cultured Jurkat cells with EdU (5-ethynyl-2′-deoxyuridine), a nucleoside analog to thymidine that is incorporated into DNA during active DNA synthesis and can be readily detected with a click reaction (Breinbauer and Kohn 2003), by means of a fluorescent dye. Thus, EdU at 10 μM was added to Jurkat cells cultured in the presence or absence of NPs at 15 μg/mL to evaluate proliferation occurring after the addition of nanoparticles. As shown in Fig. 10a (see also supplementary Fig. S.3 for individual histograms with percentages and supplementary Fig. S.4 for ungated populations), most positive control cells cultured in the absence of NP, as wells as cells cultured in the presence of NPs Au-C, Au-CYS, and Au-TC, have proliferated and thus incorporated Edu in their DNA (87.51 ± 6.17% in gate P for positive control cells and 89.64 ± 3.77%, 87.20 ± 3.01%, and 88.65 ± 3.57% in gate P for Au-C, Au-CYS, and Au-TC, respectively), while only 56.73 ± 2.54% of cells cultured in the presence of Au-GSH have proliferated. Conversely, only 8.40 ± 5.17% positive control cells cultured in the absence of NP are in gate “NP” for non-proliferating cells. Au-C, Au-CYS, and Au-TC show a similar percentage of non-proliferating cells (6.67 ± 3.34, 6.77 ± 2.43, and 7.35 ± 3.78, respectively), while 30.17 ± 8.40% of cells cultured in the presence of Au-GSH fall in the gate for non-proliferating cells, together with 98.54 ± 0.73% negative control cells that have not incorporated Edu (Fig. 10b). Thus, Au-GSH developed in the present fashion shows a significant (*p* < 0.001) antiproliferative effect towards a percentage of tumor cells.

## Conclusions

We have prepared highly soluble and stable sub-10 nm Au NPs, functionalized with different capping agents. Citrate capped gold NPs (Au-C), prepared by the classical route, do not show the expected size homogeneity, being it necessary to include modifications in the synthesis to reach the high size mono-dispersion found for Au-TC NPs. Thiol capped gold NPs (Au-CYS and Au-GSH) show a homogeneous size distribution, with a particularly small average size for GSH capped NPs where a high proportion of GSH molecules are linked to each gold core. Citrate capped gold NPs show high colloidal stability at neutral and slightly basic pH, being it possible to expand the stability range down to acidic pH for thiol capped NPs and particularly for Au-CYS NPs. All NPs have a negatively charged surface as prepared, except the Au-CYS, in which the terminal amine groups present in cysteamine, which provides a surface positive charge. These surface characteristics contribute to the poor PL emission of cysteamine capped NPs. However, Au-TC and Au-GSH NPs show relatively intense blue PL emission, particularly in the GSH capped NPs, in which the thiol-metal core transference transitions considerably enhance the fluorescent emission; however, for cellular application, their higher toxicity makes them less suitable than NP-TC. The developed NPs show a high biocompatibility with low cytotoxicity even at high concentration. It is interesting to highlight that viability and toxicity profiles have been performed not only in tumor but also in primary cells. The inclusion of an assay that specifically measures proliferation also adds in the information on the biocompatibility profile of our NPs. On the other hand, the fact that Au-GSH NPs at high concentration show toxicity and inhibit proliferation of tumor cells opens an interesting avenue of research for additional applications that we will further pursue.

## Electronic supplementary material

ESM 1 (DOCX 11933 kb)
