# Supplementary material for: Influence of size and surface capping on photoluminescence and cytotoxicity of gold nanoparticles
Source: J Nanopart Res. 2018 Nov 15;20(11):305. doi: 10.1007/s11051-018-4406-0 (PMC6244783; doi:10.1007/s11051-018-4406-0)
Supplement: Supplementary file 1 — (DOCX 11933 kb) [file 11051_2018_4406_MOESM1_ESM.docx]

**SUPPLEMENTARY MATERIAL**

| 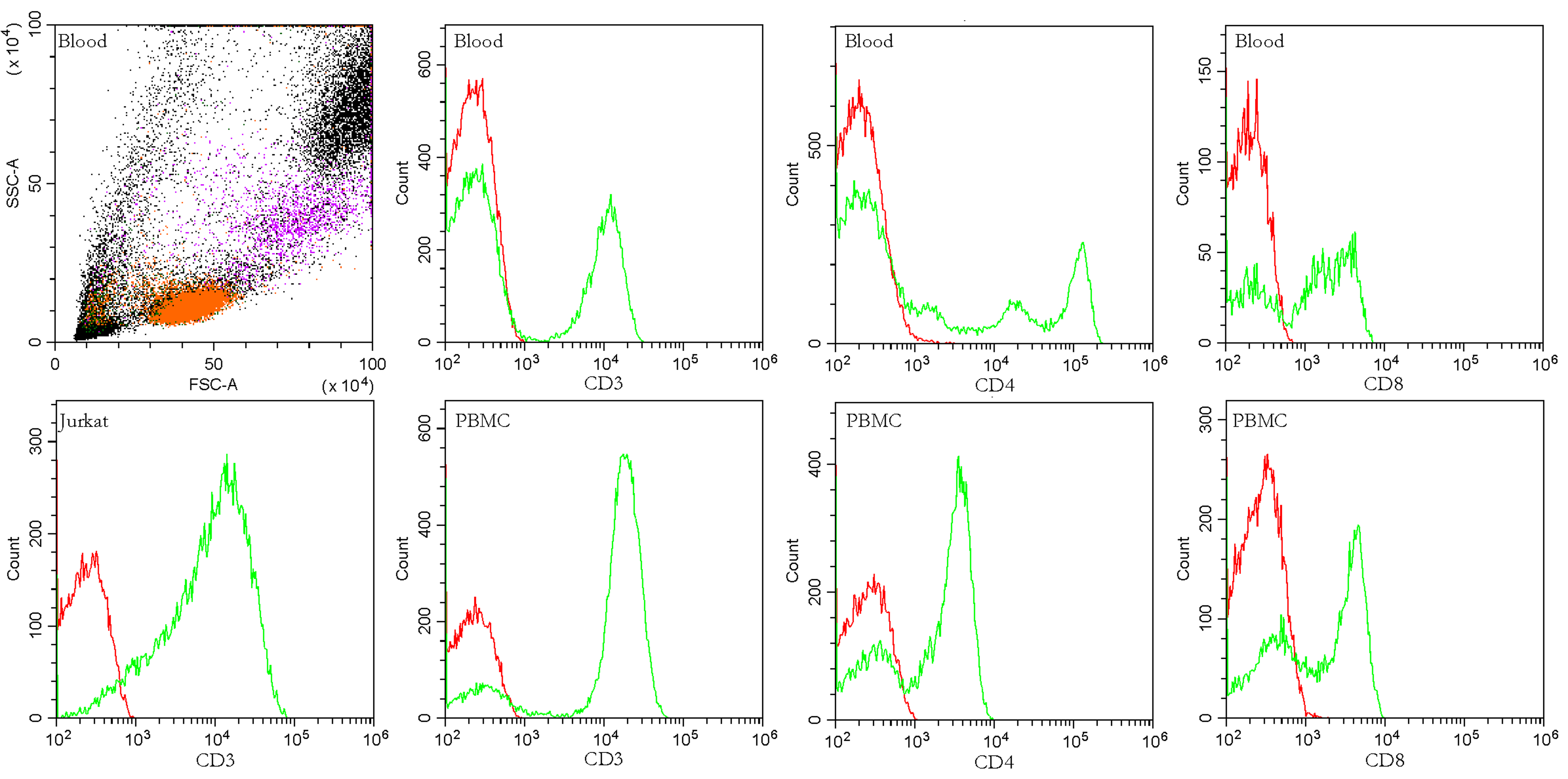 |
| --- |
| Figure S.1: FACS results showing FSC vs SSC profile as well as CD3, CD4 and CD8 staining of a blood sample were erythrocytes have been lysed with the EasyLyse™ reagent (Dako) before gradient separation (upper panels). CD3, CD4 and CD8 staining of the PBMC blasts the day of Np addition, thus 6 days after PHA stimulation (lower left three panels) and Jurkat staining with antiCD3 (lower left panel). Stained histograms are shown in green while unstained controls are shown in red. |

| Figure S.1 shows DLS size distributions obtained for Au-CYS and Au-TC NPs. In the case of Au-CYS (figure S.1.a). The DLS size distribution obtained for Au-CYS shows an average size centered at 8 nm, slightly higher than that obtained from TEM for the same sample ( 6.5 nm). In this case, TEM and DLS size differences could be attributed to CYS capping effect on the hydrodynamic diameter. The Au-TC size distribution shows an average size centered at 6.9 nm, a value similar to that obtained from TEM (7 nm). However, comparing TEM and DLS distribution for this sample (Figure 2. c and Figure S.1.b ) we can appreciate that DLS distribution shows the presence of a higher portion of NPs with sizes bigger than the maximum, that could be attribute to the influence of capping on hydrodynamic diameter. DLS distribution are slightly wider than TEM distributions (with PDI factor higher than 0.1) which can be explain the influence of capping in hydrodynamic size. |
| --- |
|  |
| Figure S.2: Size distributions obtained from DLS for Au-CYS (a) and Au-TC (b). |

| 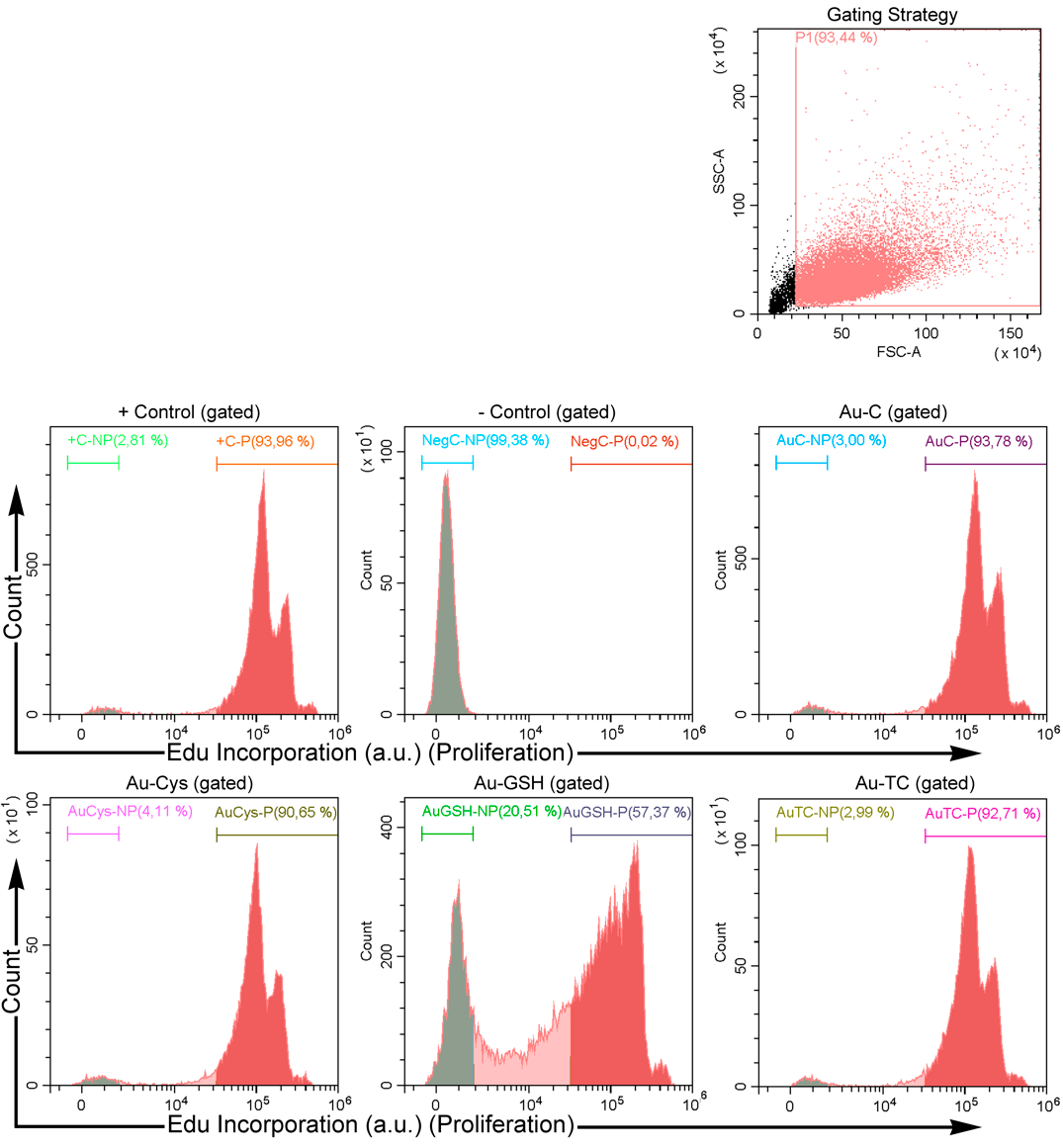 |
| --- |
| Figure S.3: Detection of proliferating cells by EdU incorporation and labeling. Jurkat tumor cells were cultured with 10μM EdU, in the presence or absence of the indicated nanoparticles at 15μg/mL. Proliferation was determined as a function of Edu incorporation into cells’ DNA, detected by means of a Pacific blue fluorescent dye. EdU+ Proliferating (gated in P) and non proliferating cells (NP) were clearly and distinctly separated by FACS. Jurkat cells in the absence of NP were used as a positive control (+ Control) for proliferation as they are a tumor cell line that spontaneously proliferate in culture. Cells that have not incorporated Edu were used as negative control (- Control). Upper pannel) Size (FSC vs. granularity (SSC) dot plot, showing the gating strategy. Middle and lower panels) Histograms showing Edu incorporation in individual samples (showing percentages for each gate), from those shown in the overlay in figure 10. |

| 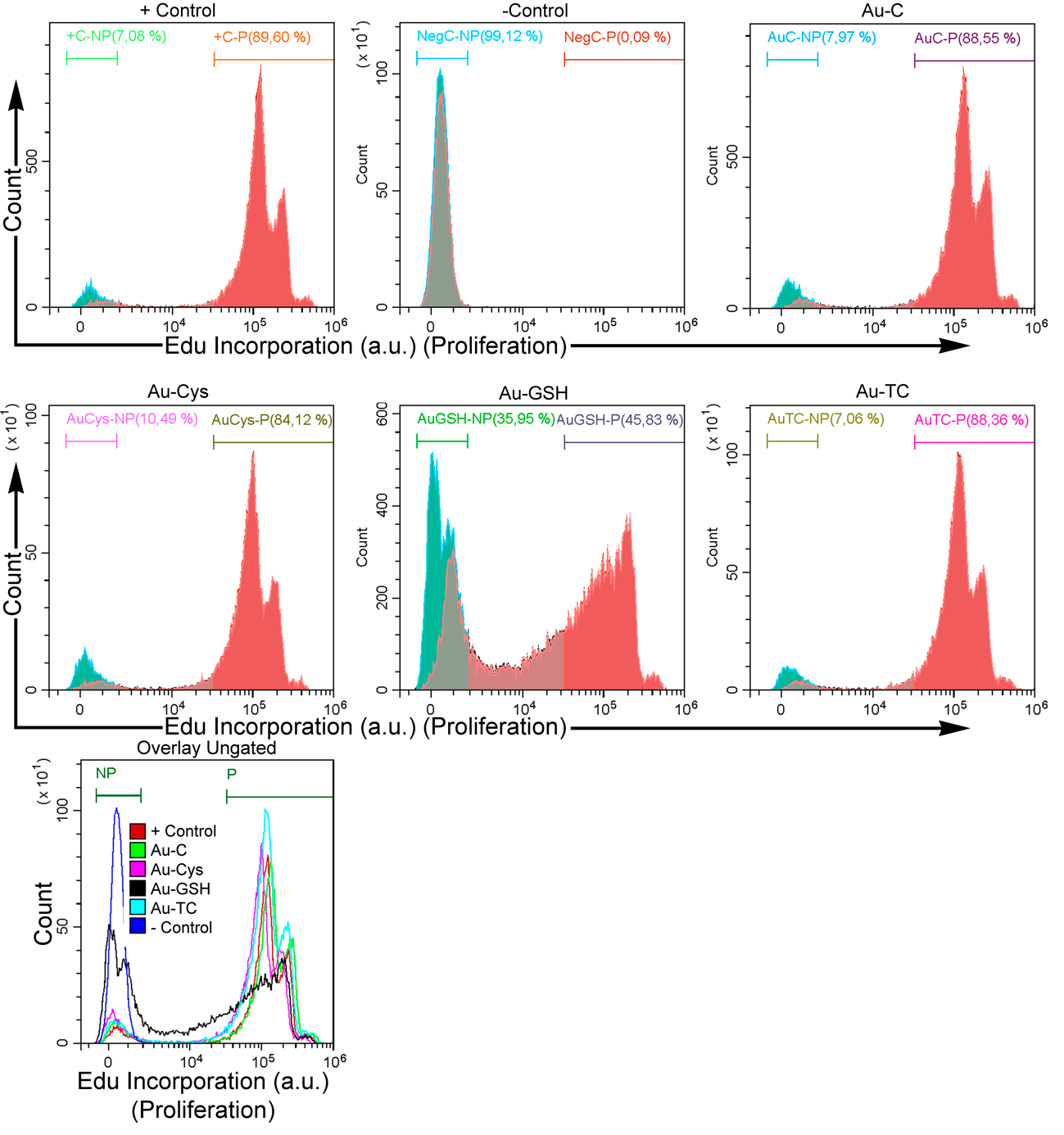 |
| --- |
| Figure S.4: An overlay histogram of the ungated populations as well as results corresponding to individual ungated samples from the experiments in figure 10. |

| **NPs** | **Z-Potential** |
| --- | --- |
| Au-C pH 7 | -158 |
| Au-C pH 10 | -143 |
| Au-TC pH 8 | -99 |
| Au-TC pH 3.5 | -33 |
| Au-CYS 3 | +88 |
| Au-CYS 7 | +21 |
| Au-GSH 9 | -140 |
| Au-GSH 7 | -31 |

Table S.1: Z-Potential values obtained for Au NPs at different pH.
